# Antiseizure Properties of Histamine H_3_ Receptor Antagonists Belonging 3,4-Dihydroquinolin-2(1*H*)-Ones

**DOI:** 10.3390/molecules28083408

**Published:** 2023-04-12

**Authors:** Yi Hua, Mingxia Song, Qiaoyue Guo, Yiqin Luo, Xianqing Deng, Yushan Huang

**Affiliations:** 1 Health Science Center, Jinggangshan University, Ji’an 343009, China; 2 Center for Evidence Based Medical and Clinical Research, First Affiliated Hospital of Gannan Medical University, Ganzhou 341000, China

**Keywords:** antiepileptics, antiseizure, anticonvulsant, H_3_R antagonists, 3,4-dihydroquinolin-2(1*H*)-one

## Abstract

H_3_R is becoming an attractive and promising target for epilepsy treatment as well as the discovery of antiepileptics. In this work, a series of 6-aminoalkoxy-3,4-dihydroquinolin-2(1*H*)-ones was prepared to screen their H_3_R antagonistic activities and antiseizure effects. The majority of the target compounds displayed a potent H_3_R antagonistic activity. Among them, compounds **2a**, **2c**, **2h**, and **4a** showed submicromolar H_3_R antagonistic activity with an IC_50_ of 0.52, 0.47, 0.12, and 0.37 μM, respectively. The maximal electroshock seizure (MES) model screened out three compounds (**2h**, **4a**, and **4b**) with antiseizure activity. Meanwhile, the pentylenetetrazole (PTZ)-induced seizure test gave a result that no compound can resist the seizures induced by PTZ. Additionally, the anti-MES action of compound **4a** fully vanished when it was administrated combined with an H_3_R agonist (RAMH). These results showed that the antiseizure role of compound **4a** might be achieved by antagonizing the H_3_R receptor. The molecular docking of **2h**, **4a**, and PIT with the H_3_R protein predicted their possible binding patterns and gave a presentation that **2h**, **4a**, and PIT had a similar binding model with H_3_R.

## 1. Introduction

Histamine, an endogenous biological amine, mediates various physiological and pathological processes by acting on four histamine receptors in the G protein-coupled receptor (GPCR) family [1]. Among them, histamine H_1_ receptors (H_1_R) and histamine H_2_ receptors (H_2_R) are related to anaphylactic reaction and the secretion of gastric acid, respectively. Histamine H4 receptors (H_4_R) are mainly involved in the human body’s immune response. Histamine H_3_ receptors (H_3_R) are mainly expressed in the central nervous system (CNS). As one of the auto-receptors, it negatively regulates the synthesis and secretion of histamine in the CNS [2]. Recently it was reported as relating to the physiological processes of sleep, awakening, appetite, and learning and memory [3,4]. H_3_R has become a potentially important target for the diseases of narcolepsy, Alzheimer’s, schizophrenia, learning and memory disorders, and epilepsy [5,6].

In particular, as an inhibitory heteroreceptor, H_3_R can affect the level of some neurotransmitters, such as serotonin, norepinephrine, γ-aminobutyric acid, and glutamate in the CNS related to epilepsy [7,8,9]. It has been proved that H_3_R antagonists can treat epilepsy by promoting the synthesis and secretion of histamine [10,11,12]. In addition, some studies have reported that H_3_R antagonists have obvious protective effects on NMDA-induced neuronal damage and cell death [13,14,15]. Therefore, increasing attention has been focused on H_3_R in epilepsy treatment.

In the last two decades, increasing antagonists or inverse agonists of H_3_R have been prepared and identified with antiseizure activity in several kinds of epileptic animal models [16,17,18]. Pitolisant (PIT), an H_3_R antagonist and inverse agonist, was approved in the EU for narcolepsy treatment. Meanwhile, it also has been subjected to clinical Phase II trials to treat photosensitive epilepsy [19]. The results showed that PIT can inhibit photosensitive epilepsy and gave a profitable electroencephalogram (EEG) performance when taken at the dose of 30 mg or 60 mg [20]. In addition, PIT displayed a powerful antiseizure activity in the electrical kindling model of epilepsy [21].

**Figure 1:**
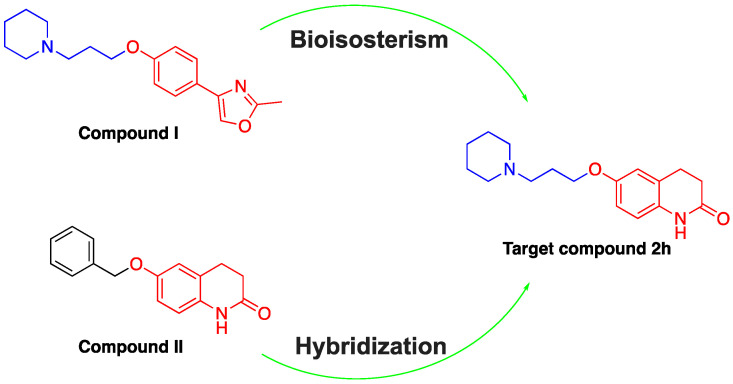
The structures of reported anticonvulsive compounds **I**, **II**, and target compound **2h**.

In our previous work, dozens of 2-methyl-4-phenyloxazoles were designed and synthesized as new H_3_R antagonists [22]. All these compounds showed micromolar to submicromolar H_3_R antagonistic activities. In addition, some of them displayed an antiseizure activity in the maximal electroshock seizure (MES) test. It is interesting to note that the antiseizure activity of the representative compound I (Figure 1, **I**) completely vanished when it was administrated combined with an H_3_R agonist R-(α)-methylhistamine (RAMH), which confirmed the correlation between H_3_R inhibition and antiepileptic activity.

To continue the program of developing antiseizure drugs from an H_3_R antagonist, herein, the 2-methyl-4-phenyloxazole moiety of the compound I was interchanged by 3,4-dihydroquinolin-2(1*H*)-one to obtain a potential H_3_R antagonist (Figure 1, compound **2h**). The 3,4-dihydroquinolin-2(1*H*)-one is an eminent pharmaceutical skeleton, which has been utilized to obtain great amounts of compounds with an antiseizure activity [23,24,25]. The hybridization of compounds **I** and **II** is expected to obtain new H_3_R antagonists with better antiseizure activity. Apart from compound **2h**, analogs (**2a-2g**, **2i**) using various kinds of amines replacing the piperidine group in compound **2h**, and derivatives (**3a-3c**, **4a-4b**) via adjusting the length of the link and introducing the substituents at the N atom of amide, were also synthesized.

All the titled compounds were synthesized smoothly and structurally confirmed by ^1^NMR, ^13^NMR, and HR-MS. Luciferase assay based on the cAMP-response element (CRE) was used to screen the H_3_R antagonism activity. Two widely used epileptic animal models, the MES model and pentylenetetrazole (PTZ)-induced seizure model, were applied to evaluate the antiseizure activity. In addition, molecular docking was carried out to understand the molecular basis of the H_3_R antagonistic activity of the prepared compounds. We hope that through this study, a new H_3_R antagonistic skeleton can be investigated to accelerate the discovery of new antiseizure drugs.

## 2. Results and Discussion

### 2.1. Chemistry

**Scheme 1:**
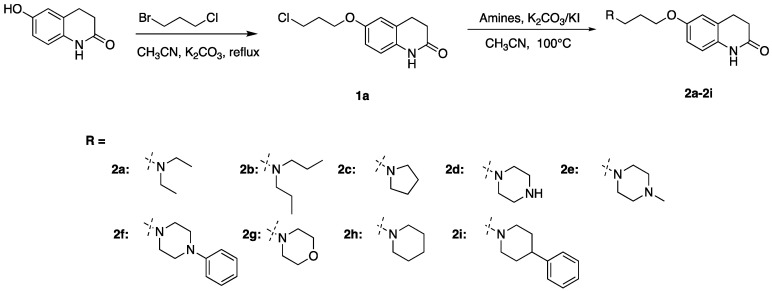
The synthesis of a first series of 3,4-dihydroquinolin-2(*1H*)-ones **2a-2i**.

**Scheme 2:**
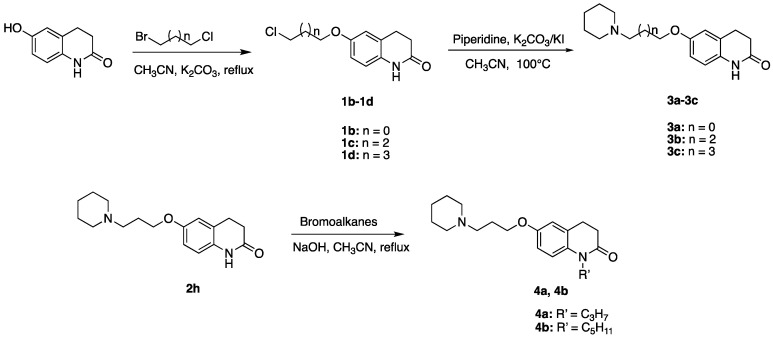
The synthesis of a second and third series of 3,4-dihydroquinolin-2(*1H*)-ones **3a-3c** and **4a-4b**.

The synthetic route of compounds **2a-2i** through a two-step route is shown in Scheme 1. Compounds **3a-3c** and **4a-4b** were prepared smoothly as shown in Scheme 2. Briefly, compounds **1a-1d** were synthesized by the reaction of 6-hydroxyquinolinone with dihaloalkane in the presence of K_2_CO_3_. The substitution reaction of compound **1a** with appropriate secondary amines gave compounds **2a-2i**. The substitution reaction of compounds **1b-1d** with piperidine provided compound **3a-3c**. Finally, compounds **4a** and **4b** were achieved by an alkylation of compound **2h**.

### 2.2. Pharmacology

#### 2.2.1. Screen for H_3_R Antagonistic Activity

CRE luciferase reporter assays are widely applied for the high-throughput screening of GPCR agonists and antagonists [26,27], which was used to screen the H_3_R antagonistic activities of the titled compounds (**2a-2i**, **3a-3c**, and **4a-4b**) in this work. PIT was used as a positive drug.

The results of targets **2a-2i**, **3a-3c**, and **4a-4b** as H_3_R antagonists are summarized in Table 1. The majority of the synthesized compounds displayed outstanding H_3_R antagonistic activities. Among them, compounds **2a** (IC_50_ = 0.52 μM), **2c** (IC_50_ = 0.47 μM), **2h** (IC_50_ = 0.12 μM), and **4a** (IC_50_ = 0.37 μM) displayed antagonistic activities at a submicromolar level. PIT showed H_3_R antagonistic activity with IC_50_ of 0.69 μM at the same condition. When the diethyl in compound **2a** was replaced by dipropyl, the H_3_R antagonistic activity decreased significantly with the IC_50_ of compound **2b** higher than 50 μM. When a phenyl group was introduced onto the piperidine of compound **2h**, the H_3_R antagonistic activity of compound **2i** declined dozens of times. This suggested that substituted tertiary amine with appropriate volume is required for the H_3_R antagonistic activity. Altering the link length of compound **2h** to obtain compounds **3a**, **3b**, and **3c**. It can be seen that the best chain length of the carbons is three, which gave the compound **2h** an IC_50_ of 0.12 μM. The H_3_R antagonistic activity decreased when the chain length became longer or shorter. Among the cyclic amine compounds, the pyrrole and piperidine substituted compounds (**2c** and **2h**) showed higher antagonistic activities than the compounds coupled with piperazine and morpholine (**2d-2g**). This may be the result of the lipophilic binding of the tertiary amine with the receptor [28]

#### 2.2.2. Evaluation of the Antiseizure Activity

To find new antiseizure candidates, the antiseizure activities of the titled compounds were evaluated via two seizure models in mice, while using PIT and antiepileptic valproic sodium (VPA) as positive controls. One of the two test models is the MES seizure model, another is the PTZ-induced seizure model. In our previous work, we found that the synthetic H_3_R antagonists and PIT showed an antiseizure activity at 10 mg/kg [22]. In addition, the administration of this dosage can minimize the impact of other possible side effects such as central inhibition and cardiac toxicity. The dose of the H_3_R antagonists **2a-2i**, **3a-3c**, **4a-4b**, and PIT was chosen as 10 mg/kg. In addition, the tested dose of VPA was 300 mg/kg.

**Figure 2:**
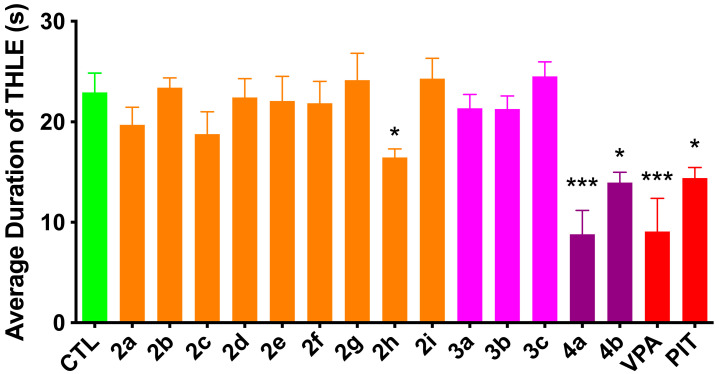
The protection of H_3_R antagonists **2a-2i**, **3a-3c**, **4a-4b** (10 mg/kg, i.p.), PIT (10 mg/kg, i.p.), and the antiepileptic drug VPA (300 mg/kg, i.p.) in the maximal electroshock seizure model. The definition of protection was the reduction or vanishing of the tonic hind limb extension (THLE). Data are shown as mean ± SEM in the figure, which are derived from seven animals in each group. * *p* < 0.05, *** *p* < 0.001 when compared to the control group (CTL).

It could be seen that all animals in the control group experienced hindlimb stiffness after the electrical stimulation in the MES model. In the treated group, the definition of protection was the reduction or vanishing of the tonic hind limb extension (THLE) in mice. As shown in Figure 2, compounds **2h**, **4a**, and **4b** as well as PIT and VPA decreased the duration of THLE significantly (*p* < 0.05, or *p* < 0.001), and showed good protection for mice. The majority of the compounds showing H_3_R antagonistic activity in Table 1 did not exhibit antiseizure activity in the MES model. However, compounds **2a**, **2c**, and **2h** hold higher H_3_R antagonistic activity and displayed a descending effect for the duration of THLE, although the effect of compounds **2a** and **2c** has no significant difference. Compounds **4a** and **4b** showed an antiseizure activity in the MES with a significant decrease in the THLE in mice. The two compounds were substituted by an alkyl group on the quinolinone, which increased their ClogP value (as seen in Table 1). This may be an important contributor to their in vivo antiseizure activity in the MES test, because marketed antiepileptics usually have a high ClogP to assure their penetration through the blood–brain barrier and run up to the site of action.

**Figure 3:**
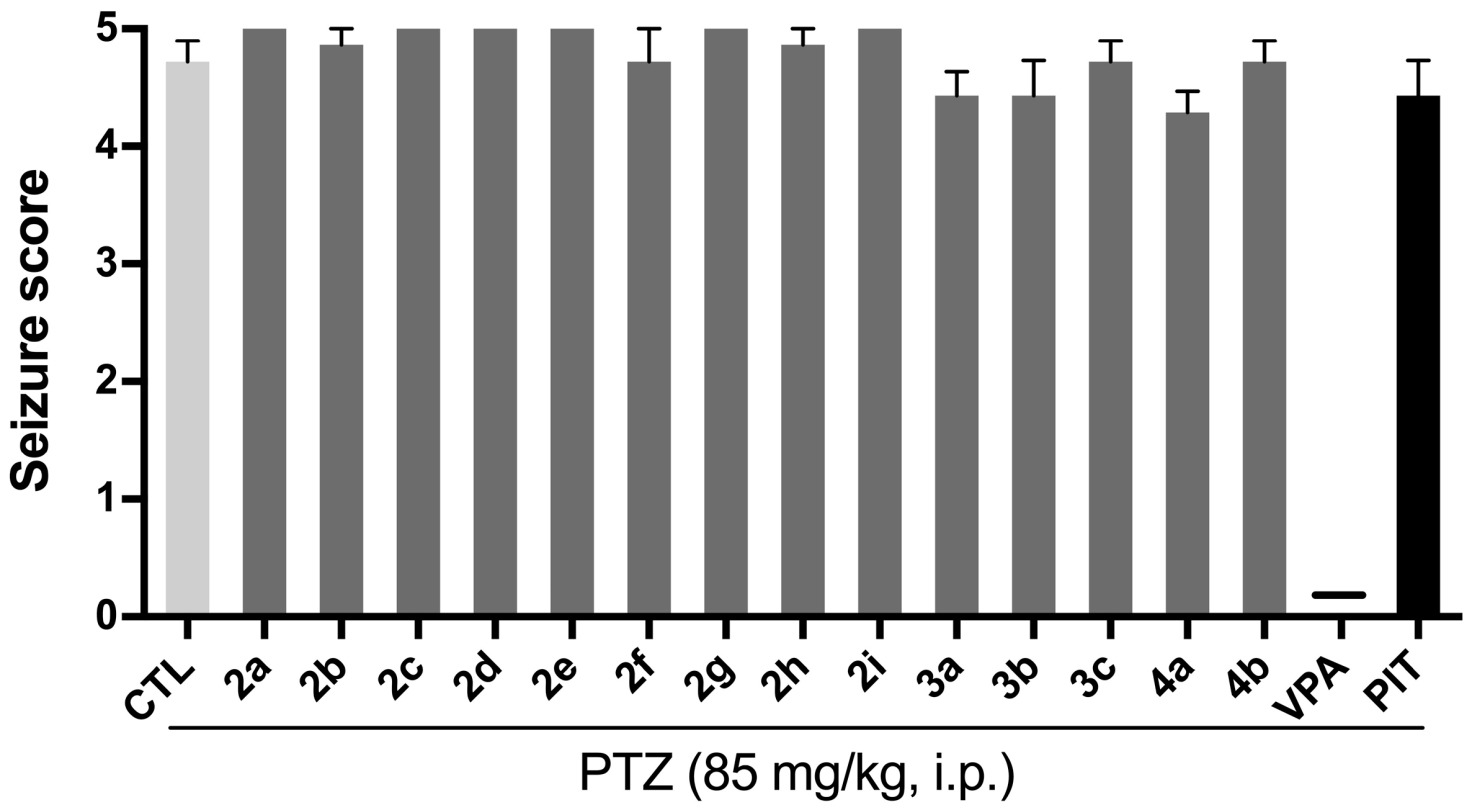
The performance of compounds **2a-2i**, **3a-3c**, and **4a-4b** (10 mg/kg, i.p.), H_3_R antagonist PIT (10 mg/kg, i.p.), and antiepileptic VPA (300 mg/kg, i.p.) in the PTZ-induced seizure model. The seizure scores came from seven animals in each group and are shown as mean ± SEM in the figure. This represents that VPA fully inhibited the convulsive behavior induced by PTZ.

The PTZ model was usually used to screen the antiepileptic candidates for the absence of seizures. In this work, compounds **2a-2i**, **3a-3c**, and **4a-4b** were also evaluated for their antiseizure action in the PTZ model in mice, while using VPA as the positive drug. As shown in Figure 3, no compound relieved the seizures induced by PTZ at the dose of 10 mg/kg (i.p.). PIT also did not exhibit protection in mice at the same dose. While VPA fully inhibited the seizure induced by PTZ when pretreated with the dosage of 300 mg/kg. The failure of synthesized compounds in the PTZ model is expected because our previously reported H_3_R inhibitors were also ineffective in the PTZ model [22]. The failure of the PIT in the PTZ model also occurred in Sadek‘s study [29]. These contradictory effects observed for the synthesized H_3_R inhibitors and PIT in the MES and PTZ models might relate to the different levels of histamine release resulting from the seizures in different seizure models [17] A considerable increase in histamine levels was found in the brain after MES-induced seizures, whereas a tendency toward a decrease in histamine levels was observed after PTZ-kindled convulsions [30].

**Figure 4:**
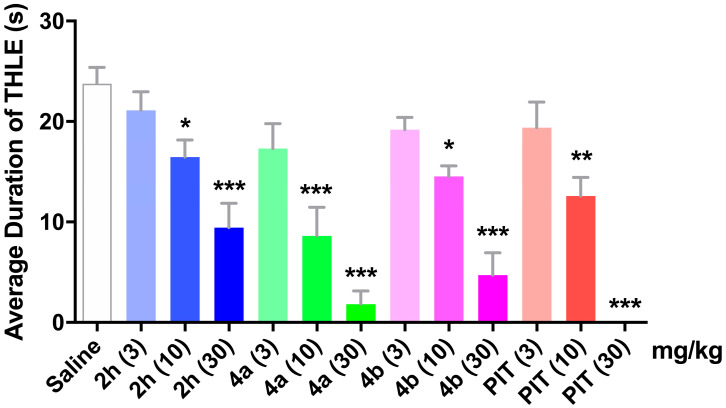
The protection of compounds **2h**, **4a**, **4b**, and PIT against the maximal electroshock-induced seizures in doses of 3, 10, and 30 mg/kg. * *p* < 0.05, ** *p* < 0.01, *** *p* < 0.001 when compared to the control group (saline).

Based on the better performance of compounds **2h**, **4a**, and **4b** in the MES seizure model, they were selected for further studies. To obtain the accurate effective dose of compounds **2h**, **4a**, and **4b** in the MES model, different dosages of three compounds were applied in the MES test. Inspiringly, the protective effect was obtained in a dose-dependent manner, although the effects at the lowest dosage (3 mg/kg) have no significant difference. As shown in Figure 4, the H_3_R antagonist PIT also exhibited a dose-dependent antiseizure activity. In particular, PIT fully abolished the THLE induced by MES at 30 mg/kg, confirming its potential antiseizure activity.

A rotarod test was carried out to estimate the neurological safety of compounds **2h**, **4a**, and **4b**. As described in Table 2, no compound showed neurotoxicity at the dose of 10 mg/kg and 30 mg/kg. Compound **2h** showed neurotoxicity with one mouse in three at 100 mg/kg, while compounds **4a**, **4b** and PIT showed no neurotoxicity at 100 mg/kg. At the dose of 300 mg/kg, all animals treated with compound **2h** and two-thirds of mice treated with compounds **4a** and **4b** were dead, which indicated that these compounds showed toxicity at the higher dose.

**Figure 5:**
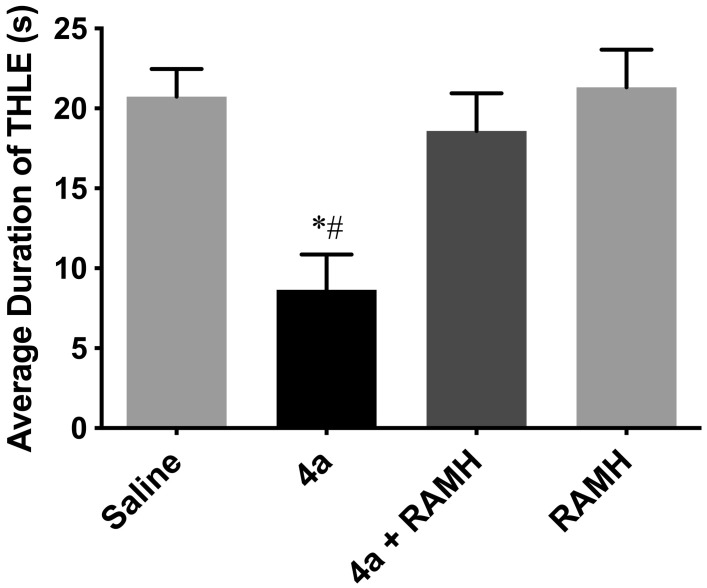
The protection of compound **4a** (10 mg/kg, i.p.) against the maximal electroshock-induced seizures in mice pretreated with RAMH (10 mg/kg, i.p.). Data come from seven animals in each group and are shown as mean ± SEM in the figure. * *p* < 0.01 when compared to the control group (saline-treated), # *p* < 0.01 when compared to the combined group (**4a** + RAMH).

Up to now, we have screened out some antiepileptic compounds from the H_3_R antagonists belonging to 3,4-dihydroquinolin-2(1*H*)-ones, especially from the molecules with a strong H_3_R inhibitory activity. However, whether their antiepileptic activity comes from their antihistamine activity is unknown. To make this clear, the ability of compound **4a** to decrease the duration of THLE in the MES model was re-evaluated in the mice pretreated by RAMH (10 mg/kg, i.p.). RAMH, as a CNS-penetrant histamine H_3_R agonist, can vanish or weaken the antihistamine effects of histamine antagonists. As shown in Figure 5, when co-injected with RAMH, compound **4a** cannot decrease the duration of THLE (*p* > 0.05). This result confirmed that the H_3_R antagonism of **4a** was an important mechanism for its antiseizure activity.

### 2.3. Molecular Docking

To make clear the molecular binding mode of the titled compounds with H_3_R and understand the molecular basis of their H_3_R antagonistic activity, the molecular docking of **2h**, **4a**, and PIT with the H_3_R protein was carried out. In this docking study, the 3D structure of H_3_R was constructed from the structure-known H_1_R protein (PDB ID: 3RZE) by homology modeling [31].

**Figure 6:**
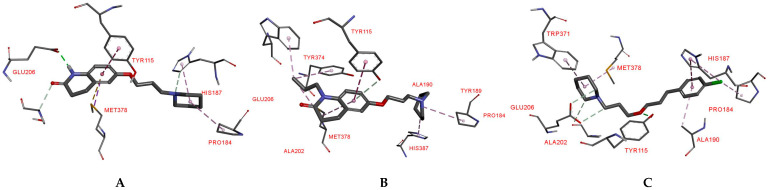
The predicted binding configurations when **2h** (**A**), **4a** (**B**), and PIT (**C**) dock with H_3_R.

**Figure 7:**
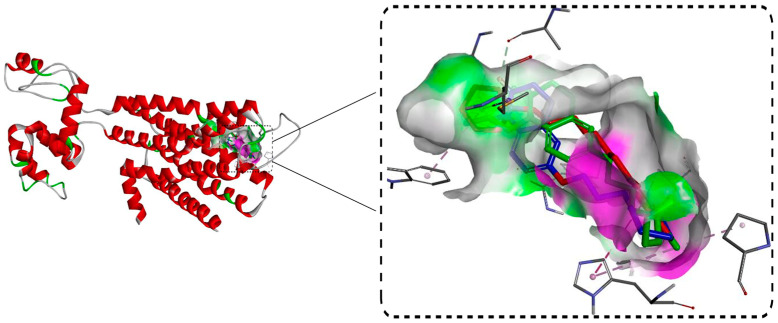
The overlying pattern of compound **2h** (blue), **4a** (green), and PIT (red) in the binding pocket of the H_3_R.

As shown in Figure 6, compounds **2h**, **4a**, and PIT bound to H_3_R in the same binding pocket and had similar binding patterns. The piperidine group in the compounds **2h** and **4a** formed a Pi–Pi interaction with the amino acid residue Pro184 of H_3_R. The phenyl group on the quinoline ring bound to the amino acid residues Tyr115 and Met378 with Pi–Pi force and alkyl interaction, respectively. The amide group in compound **2h** formed a critical H-bond interaction with Glu206 and Ser203, while this interaction vanished in the docking of compound **4a** because of the hinder of the N-substitution. This may be the important reason for the lower H_3_R antagonistic activity of **4a** than **2h**. However, the propyl group in compound **4a** formed hydrophobic interactions with Tyr374 and Met378. PIT and H_3_R interacted through the similar amino acid residues Tyr115, Pro184, His187, Ala190, Ala202, Glu206, Trp371, Met378, and so on. The overlying pattern of compounds **2h**, **4a**, and PIT was shown in Figure 7, which vividly presented that **2h**, **4a**, and PIT had a similar binding model with H_3_R. The docking score for compound **2h** and H_3_R protein binding pocket was obtained to be 115.14, which is higher than that of compound **4a** and PIT with a score of 109 and 103.19, respectively. The docking score is consistent with their antihistamine effects. These results supported the suggestion that compounds **2h** and **4a** play their antiseizure effects by binding and inhibiting the H_3_R receptor, with a similar binding model to PIT.

## 3. Materials and Methods

### 3.1. Chemical Part

Unless otherwise specified, the reagents used in this work were bought from Macklin Inc. All the reactions were monitored by thin-layer chromatography (TLC). After purification, the products were sent to the analysis center for a structure confirmation. The NMR spectrums were measured on a Bruker AV-300 spectrometer. The HR-MS of compounds was measured on a Xevo G2-XS QTOF mass spectrometer.

#### 3.1.1. Synthesis Procedure of 6-(Chloroalkoxy)-3,4-dihydroquinolin-2(1*H*)-one (**1a-1d**)

Taking compound **1a** as an example: 6-hydroxyquinolinone (1.63 g, 10 mmol), 1-bromo-3-chloropropane (1.87 g, 12 mmol), and potassium carbonate (2.76 g, 20 mmol) were added into a round-bottomed flask with 20 mL acetonitrile. After refluxing the mixture for 24 h, the finish of the reaction was identified by the TLC monitoring with 25% ethyl acetate in petroleum ether. Then the solvent was removed and the leavings were purified using silica gel column chromatography (1% methanol in DCM) to obtain the compound **1a**. The compounds **1b-1d** were obtained according to the above method using the other haloalkanes. The characterization for the four compounds is listed below.

6-(3-Chloropropoxy)-3,4-dihydroquinolin-2(1*H*)-one **(1a)** Mp 99–102 °C, yield 79%. ^1^H-NMR (CDCl_3_, 300 MHz): *δ* 2.20–2.27 (m, 2H, OCH_2_CH_2_), 2.63 (t, 2H, *J* = 8.5 Hz, CH_2_), 2.95 (t, 2H, *J* = 8.5 Hz, CH_2_), 3.75 (t, 2H, *J* = 6.3 Hz, ClCH_2_), 4.09 (t, 2H, *J* = 6.3 Hz, OCH_2_), 6.72–6.81 (m, 3H, Ph-H), 9.32 (s, 1H, CONH). ^13^C-NMR (DMSO-*d*_6_, 75 MHz): *δ* 171.9, 154.7, 131.1, 125.0, 116.4, 114.5, 113.2, 64.7, 41.5, 32.3, 30.6, 25.7. HR-MS (ESI) calcd for C_12_H_15_ClNO_2_^+^ ([M + H]^+^): 240.0786; found: 240.0791.

6-(2-Chloroethoxy)-3,4-dihydroquinolin-2(1*H*)-one (**1b**) Mp 151–152 °C, yield 81%. ^1^H-NMR (CDCl_3_, 300 MHz): *δ* 2.63 (t, 2H, *J* = 8.6 Hz, CH_2_), 2.96 (t, 2H, *J* = 8.5 Hz, CH_2_), 3.81 (t, 2H, *J* = 5.8 Hz, ClCH_2_), 4.21 (t, 2H, *J* = 5.8 Hz, OCH_2_), 6.76–6.78 (m, 3H, Ph-H), 8.76 (s, 1H, CONH). ^13^C-NMR (DMSO-*d*_6_, 75 MHz): *δ* 171.5, 154.1, 131.5, 125.2, 116.2, 115.0, 113.5, 68.6, 41.9, 30.6, 25.7. HR-MS (ESI) calcd for C_11_H_13_ClNO_2_^+^ ([M + H]^+^): 226.0629; found: 226.0628.

6-(4-Chlorobutoxy)-3,4-dihydroquinolin-2(1*H*)-one (**1c**) Mp 146–148 °C, yield 71%. ^1^H-NMR (CDCl_3_, 300 MHz): *δ* 1.91–1.99 (m, 4H, OCH_2_(CH_2_)_2_), 2.61 (t, 2H, *J* = 7.6 Hz, CH_2_), 2.93 (t, 2H, *J* = 7.7 Hz, CH_2_), 3.62 (t, 6H, *J* = 5.8 Hz, N-CH_2_), 3.96 (t, 2H, *J* = 6.2 Hz, O-CH_2_), 6.69–6.75 (m, 3H, Ph-H), 8.85 (s, 1H, CONH). ^13^C-NMR (CDCl_3_, 75 MHz): *δ* 171.6, 154.9, 130.9, 125.0, 116.3, 114.5, 113.1, 67.4, 44.7, 30.6, 29.3, 26.7, 25.7. HR-MS (ESI) calcd for C_13_H_17_ClNO_2_^+^ ([M + H]^+^): 254.0942; found: 254.0947.

6-((5-Chloropentyl)oxy)-3,4-dihydroquinolin-2(1*H*)-one (**1d**) Mp 128–130 °C, yield 72%. ^1^H-NMR (CDCl_3_, 300 MHz): *δ* 1.59–1.93 (m, 6H, OCH_2_(CH_2_)_3_), 2.62 (t, 2H, *J* = 7.6 Hz, COCH_2_), 2.93 (t, 2H, *J* = 7.6 Hz, PhCH_2_), 3.56 (t, 2H, *J* = 5.9 Hz, ClCH_2_), 3.93 (t, 2H, *J* = 6.2 Hz, OCH_2_), 6.69–6.81 (m, 3H, Ph-H), 9.51 (s, 1H, CONH). ^13^C-NMR (DMSO-*d*_6_, 75 MHz): *δ* 172.0, 154.9, 130.9, 124.9, 116.4, 114.4, 113.1, 68.0, 44.9, 32.3, 30.6, 28.6, 25.7, 23.5. HR-MS (ESI) calcd for C_14_H_19_ClNO_2_^+^ ([M + H]^+^): 268.1099; found: 268.1106.

#### 3.1.2. The Synthesis Procedure of 6-(3-(Substituted-aminopropoxy)-3,4-dihydroquinolin-2(1*H*)-one (**2a-2i**) and Its Analogues (**3a-3c**)

Taking compound **2a** as an example: compounds **1a** (1.20 g, 5 mmol), diethylamine (0.73g, 10 mmol), K_2_CO_3_ (1.38 g, 10 mmol), and KI (1.0 g, 6 mmol) were added to a round-bottomed flask with 20 mL acetonitrile. After refluxing the mixture for 24 h, the finish of the reaction was confirmed by the TLC monitoring with 50% ethyl acetate in a petroleum ether. After cooling to room temperature, the mixture was filtered, and the filtrate was evaporated to obtain a crude product, which was purified on silica gel column chromatography (1.5% MeOH in DCM) to obtain compound **2a**. Compounds **2b-2i** were prepared according to the above method with the compound **1a** and other secondary amines. The compounds **3a** (**3b**, **3c**) were prepared according to the above method with the compounds **1b** (**1c**, **1d**) and piperidine. The characterization for these compounds is listed below.

6-(3-(Diethylamino)propoxy)-3,4-dihydroquinolin-2(1*H*)-one hydrochioride **(2a)** Mp 68–70 °C, yield 76%. ^1^H-NMR (DMSO-*d*_6_, 300 MHz): *δ* 1.23 (t, 6H, *J* = 7.6 Hz, CH_3_), 2.08–2.15 (m, 2H, CH_2_), 2.38 (t, 2H, *J* = 7.5 Hz, CH_2_), 2.82, (t, 2H, *J* = 7.5 Hz, CH_2_), 3.05–3.17 (m, 6H, N-CH_2_), 4.00 (t, 2H, *J* = 6.0 Hz, O-CH_2_), 6.72–6.81 (m, 3H, Ph-H), 9.93 (s, 1H, CONH), 10.81 (s, 1H, HCl). ^13^C-NMR (DMSO-*d*_6_, 75 MHz): *δ* 170.21, 153.36, 132.46, 125.34, 116.25, 114.65, 113.53, 65.64, 46.57, 43.13, 30.79, 25.53, 23.54, 8.87. HR-MS (ESI) calcd for C_16_H_25_N_2_O_2_^+^ ([M + H]^+^): 277.1911; found: 277.1912.

6-(3-(Dipropylamino)propoxy)-3,4-dihydroquinolin-2(*1H*)-one hydrochloride **(2b)** Mp 57–59 °C, yield 71%. ^1^H-NMR (DMSO-*d*_6_, 300 MHz): *δ* 0.96 (t, 6H, *J* = 7.5 Hz, CH_3_), 1.71–1.84 (m, 4H, CH_2_), 2.17–2.22 (m, 2H, CH_2_), 2.43 (t, 2H, *J* = 7.5 Hz, CH_2_), 2.84 (t, 2H, *J* = 7.5 Hz, CH_2_), 2.98–3.05 (m, 4H, N-CH_2_), 3.20–3.26 (m, 2H, N-CH_2_), 3.99 (t, 2H, *J* = 5.6 Hz, O-CH_2_), 6.61–6.79 (m, 3H, Ph-H), 9.84 (s, 1H, CONH), 10.73 (s, 1H, HCl). ^13^C-NMR (DMSO-*d*_6_, 75 MHz): *δ* 175.10, 158.50, 137.05, 129.70, 121.09, 119.07, 118.00, 70.02, 59.02, 54.72, 35.56, 30.48, 28.39, 21.78, 16.03. HR-MS (ESI) calcd for C_18_H_29_N_2_O_2_^+^ ([M + H]^+^): 305.2224; found: 305.2223.

6-(3-(Pyrrolidin-1-yl)propoxy)-3,4-dihydroquinolin-2(*1H*)-one hydrochioride **(2c)** Mp 120–122 °C, yield 83%. ^1^H-NMR (DMSO-*d*_6_, 300 MHz): *δ* 1.86–2.07 (m, 6H, CH_2_), 2.40 (t, 2H, *J* = 7.5 Hz, CH_2_), 2.84 (t, 2H, *J* = 7.5 Hz, CH_2_), 3.05 (s, 2H, N-CH_2_), 3.33 (s, 2H, N-CH_2_), 3.59 (s, 2H, N-CH_2_), 4.00 (s, 2H, *J* = 6.0 Hz, O-CH_2_), 6.3–6.80 (m, 3H, Ph-H), 9.44 (s, 1H, CONH), 9.90 (s, 1H, HCl). ^13^C-NMR (DMSO-*d*_6_, 75 MHz): *δ* 170.22, 153.79, 132.50, 125.34, 116.24, 114.60, 113.59, 65.41, 53.89, 51.94, 30.80, 25.89, 25.55, 23.06. HR-MS (ESI) calcd for C_16_H_23_N_2_O_2_^+^ ([M + H]^+^): 275.1754; found: 275.1755.

6-(3-(Piperazin-1-yl)propoxy)-3,4-dihydroquinolin-2(*1H*)-one **(2d)** Mp 150–151 °C, yield 78%. ^1^H-NMR (DMSO-*d*_6_, 300 MHz): *δ* 1.77–1.86 (m, 2H, CH_2_), 2.37–2.51 (m, 12H, CH_2_), 2.83 (t, 2H, *J* = 7.2 Hz, N-CH_2_), 3.92 (t, 2H, *J* = 6.1 Hz, O-CH_2_), 6.69–6.76 (m, 3H, Ph-H), 9.86 (s, 1H, N-H). ^13^C-NMR (DMSO-*d*_6_, 75 MHz): *δ* 170.19, 154.28, 132.15, 125.28, 116.23, 114.48, 113.45, 66.53, 55.15, 53.11, 46.11, 30.84, 26.76, 25.57, HR-MS (ESI) calcd for C_16_H_24_N_3_O_2_^+^ ([M + H]^+^): 290.1863; found: 290.1864.

6-(3-(4-Methylpiperazin-1-yl)propoxy)-3,4-dihydroquinolin-2(1*H*)-one **(2e)** Mp 159–160 °C, yield 79%. ^1^H-NMR (DMSO-*d*_6_, 300 MHz): *δ* 1.49–1.67 (m, 7H, CH_2_, NCH_3_), 1.95–2.05 (m, 2H, CH_2_), 2.40 (t, 2H, *J* = 7.6 Hz, N-CH_2_), 2.81–2.89 (m, 8H, N-CH_2_), 3.97 (t, 2H, *J* = 6.2 Hz, O-CH_2_), 6.70–6.79 (m, 3H, Ph-H), 9.89 (s, 1H, CONH). ^13^C-NMR (DMSO-*d*_6_, 75 MHz): *δ* 170.19, 153.98, 132.39, 125.32, 116.24, 114.57, 113.56, 65.98, 54.54, 53.35, 30.82, 25.56, 25.01, 24.13, 22.75. HR-MS (ESI) calcd for C_17_H_26_N_3_O_2_^+^ ([M + H]^+^): 304.2020; found: 304.2015.

6-(3-(4-Phenylpiperazin-1-yl)propoxy)-3,4-dihydroquinolin-2(1*H*)-one **(2f)** Mp 199–201 °C, yield 80%. ^1^H-NMR (DMSO-*d*_6_, 300 MHz): *δ* 1.89 (t, 2H, *J* = 6.2 Hz, CH_2_), 2.37–2.51 (m, 8H, CH_2_), 2.83 (t, 2H, *J* = 7.3 Hz, CH_2_), 3.12 (s, 4H, CH_2_), 3.96 (t, 2H, *J* = 6.2 Hz, O-CH_2_), 6.75–6.79 (m, 4H, Ph-H), 6.92 (d, 2H, *J* = 7.2 Hz, Ph-H), 7.21 (t, 2H, *J* = 8.6 Hz, Ph-H), 9.89 (s, 1H, CONH). ^13^C-NMR (DMSO-*d*_6_, 75 MHz): *δ* 170.20, 154.28, 151.51, 132.17, 129.36, 125.29, 119.20, 116.23, 115.77, 114.50, 113.46, 66.54, 54.95, 53.27, 48.67, 30.84, 26.76, 25.57. HR-MS (ESI) calcd for C_22_H_28_N_3_O_2_^+^ ([M + H]^+^): 366.2176; found: 366.2177.

6-(3-(Morpholino)propoxy)-3,4-dihydroquinolin-2(1*H*)-one **(2g)** Mp 146–147 °C, yield 79%. ^1^H-NMR (DMSO-*d*_6_, 300 MHz): *δ* 1.83–1.92 (m, 2H, CH_2_), 2.40–2.46 (m, 8H, CH_2_), 2.86 (t, 2H, *J* = 7.3 Hz, N-CH_2_), 3.62 (t, 4H, *J* = 4.5 Hz, N-CH_2_), 3.94 (t, 2H, *J* = 6.2 Hz, O-CH_2_), 6.64–6.79 (m, 3H, Ph-H), 9.85 (s, 1H, N-H). ^13^C-NMR (DMSO-*d*_6_, 75 MHz): *δ* 170.19, 154.26, 132.15, 125.28, 116.22, 114.46, 113.43, 66.67, 66.45, 55.36, 53.85, 30.83, 26.42, 25.56. HR-MS (ESI) calcd for C_16_H_23_N_2_O_3_^+^ ([M + H]^+^): 291.1703; found: 291.1702.

6-(3-(Piperidin-1-yl)propoxy)-3,4-dihydroquinolin-2(1*H*)-one **(2h)** Mp 138–140 °C, yield 79%. ^1^H-NMR (DMSO-*d*_6_, 300 MHz): *δ* 1.78–1.87 (t, 2H, CH_2_), 2.38 (t, 2H, *J* = 7.4 Hz, CH_2_), 2.47–2.60 (m, 6H, CH_2_), 2.60 (s, 4H, CH_2_), 2.82 (t, 2H, *J* = 7.4 Hz, CH_2_), 3.09 (s, 4H, N-CH_2_), 3.24 (s, 2H, N-CH_2_), 3.93 (t, 2H, *J* = 6.2 Hz, O-CH_2_), 6.69–6.76 (m, 3H, Ph-H), 9.89 (s, 1H, CONH). ^13^C-NMR (DMSO-*d*_6_, 75 MHz): *δ* 170.20, 154.19, 132.18, 125.29, 116.23, 114.51, 113.49, 66.29, 54.48, 49.76, 43.43, 41.15, 30.83, 26.40, 25.56. HR-MS (ESI) calcd for C_17_H_25_N_2_O_2_^+^ ([M + H]^+^): 289.1911; found: 289.1912.

6-(3-(4-Phenylpiperidin-1-yl)propoxy)-3,4-dihydroquinolin-2(1*H*)-one **(2i)** Mp 174–176 °C, yield 78%. ^1^H-NMR (DMSO-*d*_6_, 300 MHz): *δ* 1.27 (s, 1H, CH), 1.79–2.03 (m, 6H, CH_2_), 2.30 (s, 2H, *J* = 6.2 Hz, CH_2_), 2.49–2.68 (m, 4H, N-CH_2_), 2.89 (t, 2H, *J* = 6.2 Hz, N-CH_2_), 3.16 (d, 2H, *J* = 7.9 Hz, N-CH_2_), 4.00 (s, 2H, *J* = 6.2 Hz, O-CH_2_), 6.66–6.81 (m, 3H, Ph-H), 7.18–7.31 (m, 5H, Ph-H), 9.67 (s, 1H, CONH). ^13^C-NMR (DMSO-*d*_6_, 75 MHz): *δ* 170.4, 154.2, 145.7, 131.7, 128.4, 126.7, 126.1, 124.7, 116.1, 114.1, 113.1, 66.2, 55.0, 53.9, 41.8, 32.8, 30.7, 26.3, 25.7. HR-MS (ESI) calcd for C_23_H_29_N_2_O_2_^+^ ([M + H]^+^): 365.2224; found: 365.2223.

6-(2-(Piperidin-1-yl)ethoxy)-3,4-dihydroquinolin-2(1*H*)-one **(3a)** Mp 147–149 °C, yield 77%. ^1^H-NMR (CDCl_3_, 300 MHz): *δ* 1.66–1.97 (m, 8H, CH_2_), 2.50 (t, 2H, *J* = 7.3 Hz, CH_2_), 2.89 (t, 2H, *J* = 7.3 Hz, CH_2_), 3.10 (t, 2H, *J* = 6.0 Hz, CH_2_), 3.58 (t, 2H, *J* = 4.7 Hz, CH_2_), 4.38 (t, 2H, *J* = 4.7 Hz, O-CH_2_), 6.70–6.86 (m, 3H, Ph-H), 9.64 (s, 1H, CONH). ^13^C-NMR (CDCl_3_, 75 MHz): *δ* 175.40, 157.54, 137.55, 129.85, 121.19, 119.28, 118.24, 67.72, 60.80, 58.61, 49.32, 35.43, 30.44, 27.69. HR-MS (ESI) calcd for C_16_H_23_N_2_O_2_^+^ ([M + H]^+^): 275.1754; found: 275.1756.

6-(4-(Piperidin-1-yl)butoxy)-3,4-dihydroquinolin-2(1*H*)-one **(3b)** Mp 150–151 °C, yield 78%. ^1^H-NMR (CDCl_3_, 300 MHz): *δ* 1.58–1.68 (m, 2H, CH_2_), 1.82–2.01 (m, 8H, CH_2_), 2.62 (t, 2H, *J* = 7.5 Hz, CH_2_), 2.75–2.85 (m, 6H, CH_2_), 2.96 (t, 2H, *J* = 7.5 Hz, N-CH_2_), 3.99 (t, 2H, *J* = 6.1 Hz, O-CH_2_), 6.67–6.74 (m, 3H, Ph-H), 7.70 (s, 1H, CONH). ^13^C-NMR (CDCl_3_, 75 MHz): *δ* 170.83, 154.74, 130.82, 125.16, 115.97, 114.69, 113.20, 67.70, 58.00, 53.80, 30.64, 27.00, 25.74, 23.91, 23.06, 21.99. HR-MS (ESI) calcd for C_18_H_27_N_2_O_2_^+^ ([M + H]^+^): 303.2067; found: 303.2068.

6-((5-(Piperidin-1-yl)pentyl)oxy)-3,4-dihydroquinolin-2(1*H*)-one **(3c)** Mp 150–151 °C, yield 79%. ^1^H-NMR (CDCl_3_, 300 MHz): *δ* 1.53–1.63 (m, 4H, CH_2_), 1.80–1.89 (q, 4H, CH_2_), 2.00–2.11 (m, 6H, CH_2_), 2.62 (t, 2H, *J* = 7.4 Hz, CH_2_), 2.93–3.01 (m, 6H, -NCH_2_), 3.97 (t, 2H, *J* = 6.2 Hz, 0-CH_2_), 6.66–6.76 (m, 3H, Ph-H), 7.61 (s, 1H, N-H). ^13^C-NMR (CDCl_3_, 75 MHz): *δ* 170.76, 164.81, 130.76, 125.17, 116.93, 114.67, 113.23, 67.66, 57.39, 53.26, 30.66, 28.66, 25.74, 23.67, 23.34, 22.36, 22.02. HR-MS (ESI) calcd for C_19_H_29_N_2_O_2_^+^ ([M + H]^+^): 317.2224; found: 317.2222.

#### 3.1.3. Synthesis Procedure of 1-Alkyl-6-(3-(piperidin-1-yl)propoxy)-3,4-dihydroquinolin-2(1H)-one (**4a-4b**)

Sodium hydroxide (0.12 g, 3 mmol), compound **2h** (0.6 g, 2 mmol), and bromopropane (0.37 g, 3 mmol) were placed into a flask with 20 mL of acetonitrile, and the mixture was refluxed for 24 h. Then the brine was added after evaporating the solvent of the mixture. The combined organic layer was dried over anhydrous MgSO_4_ after the extraction with DCM 3 times (30 mL × 3). A crude product was obtained after filtration and evaporation. The residue was purified by silica gel column chromatography with 1% methanol in DCM to obtain the compound **4a**. The bromopropane was replaced by pentane bromide to give the compound **4b**. The characterization for the two compounds is listed below.

6-(3-(Piperidin-1-yl)propoxy)-1-propyl-3,4-dihydroquinolin-2(1*H*)-one **(4a)** oil, yield 78%. ^1^H-NMR (CDCl_3_, 300 MHz): *δ* 0.87 (t, 3H, J = 7.5 Hz, CH_3_), 1.51–1.63 (m, 4H, CH_2_), 1.77–1.85 (m, 4H, CH_2_), 2.12–2.21 (m, 2H, CH_2_), 2.52 (t, 2H, *J* = 7.4 Hz, CH_2_), 2.75–2.85 (m, 8H, CH_2_), 3.78 (t, 2H, *J* = 7.5 Hz, N-CH_2_), 3.96 (t, 2H, *J* = 6.1 Hz, O-CH_2_), 6.65–6.83 (m, 3H, Ph-H). ^13^C-NMR (CDCl_3_, 75 MHz): *δ* 169.63, 154.01, 133.25, 128.12, 115.77, 114.49, 112.61, 65.92, 55.49, 53.91, 43.56, 31.34, 25.76, 25.14, 23.97, 22.97, 20.34, 11,14. HR-MS (ESI) calcd for C_20_H_31_N_2_O_2_^+^ ([M + H]^+^): 331.2380; found: 331.2381.

1-Pentyl-6-(3-(piperidin-1-yl)propoxy)-3,4-dihydroquinolin-2(1*H*)-one **(4b)** oil, yield 77%. ^1^H-NMR (CDCl_3_, 300 MHz): *δ* 0.90 (t 3H, *J* = 7.1 Hz, CH_3_), 1.29–1.72 (m, 10H, CH_2_), 2.13 (s, 4H, CH_2_), 2.44–2.63 (m, 4H, CH_2_), 2.84 (t, 2H, *J* = 6.2 Hz, N-CH_2_), 3.18–3.23 (m, 4H, N-CH_2_), 3.88 (t, 2H, *J* = 7.5 Hz, N-CH_2_), 4.07 (t, 2H, *J* = 4.8 Hz, O-CH_2_), 6.70–6.91 (m, 3H, Ph-H). ^13^C-NMR (CDCl_3_, 75 MHz): *δ* 169.61, 153.66, 133.67, 128.31, 115.83, 114.51, 112.58, 65.35, 55.40, 53.57, 42.17, 31.85, 29.03, 26.88, 25.83, 23.99, 22.55, 22.43, 22.07, 14.01. HR-MS (ESI) calcd for C_22_H_35_N_2_O_2_^+^ ([M + H]^+^): 359.2693; found: 359.2694.

### 3.2. Pharmacological Assays

#### 3.2.1. Luciferase Assay for the H_3_R Antagonistic Activity

Luciferase assay was taken to screen the H_3_R antagonistic activity of the compounds **2a-2i**, **3a-3c**, and **4a-4b**. PIT was used as the positive control. Firstly, all the target compounds were screened at 5 μM and 50 μM. In addition, the compounds with an inhibitory rate bigger than 50% were subjected to further assay to obtain the IC_50_. The detailed experimental operations refer to the previous paper [22,31].

#### 3.2.2. In Vivo Pharmacology Tests for the Antiseizure Activity

The antiseizure activity of the compounds **2a-2i**, **3a-3c**, and **4a-4b** was examined through the MES and PTZ models. The neurological safety of the compounds **2h**, **4a**, and **4b** was evaluated by the rotarod test. In the MES test, the definition of protection was the reduction or vanishing of the THLE in mice. In the PTZ test, the seizures were induced through the subcutaneous administration of PTZ (85 mg/kg). The antiseizure activities of the compounds in the PTZ test were evaluated by comparing the convulsion scores in mice between taking the compounds or not. VPA and PIT were used as positive drugs. To explore the anticonvulsive mechanism, compound **4a** (10 mg/kg) was co-injected with RAMH (10 mg/kg). In addition, the THLE was recorded and compared with that of the mice treated by compound **4a** alone. The detailed experimental operations in the MES, PTZ, and rotarod tests refer to our previous paper [22,32]. All the animal experiments were carried out on 4-week-old KunMing male mice weighing from 20 to 25 g. The mice were housed collectively in polycarbonate cages in groups of 30, where they were maintained on a 12 h light/dark cycle in a temperature controlled (25 ± 2 °C) laboratory with free access to food and water. In the MES and PTZ models, seven animals were used in each group. In the rotarod test, three animals were used in each group. Each animal was used only once. All the experiments referring to animals were applied according to the Guide for the Care and Use of Laboratory Animals and were approved by the Medical Ethics Committee of Jinggangshan University, approval number: 220811002.

#### 3.2.3. Statistics

GraphPad Prism was used to carry out the statistical analysis. In the seizure tests, data were provided as the mean ± standard error of the mean (SEM). In addition, one-way ANOVA followed by Dunnett’s post hoc test was executed for group comparison. The statistical significance was defined when the *p* value < 0.05 [22].

#### 3.2.4. Molecular Docking

The molecular docking was conducted through the Flexible Docking in Discovery Studio 2019 [22]. The 3D structure of H_3_R was constructed from the structure-known H_1_R protein (PDB ID: 3RZE) by homology modeling [30]. The 3D structures of the compounds **2h**, **4a**, and PIT were constructed by ChemDraw 16.0, energy minimized, and placed into the protein cavity to flexible binding with the homology model of H_3_R. After the docking running finished, the binding patterns were provided, and the interactions between the compounds **2h**, **4a**, PIT, and H_3_R were analyzed.

## 4. Conclusions

In this work, a battery of 6-aminoalkoxy-3,4-dihydroquinolin-2(1*H*)-one derivatives were synthesized to screen their H_3_R antagonistic activities and evaluate their antiseizure activities. The majority of the target compounds displayed a potent H_3_R antagonistic activity. Among them, the compounds **2a**, **2c**, **2h**, and **4a** showed a submicromolar H_3_R antagonistic activity. The MES model screened out three compounds (**2h**, **4a**, and **4b**) with antiseizure activity, while all compounds did not show any protective effect for mice against the seizures induced by PTZ. Additionally, the antiseizure mechanism of compound **4a** was proved to be related to H_3_R antagonism. The molecular docking of **2h**, **4a**, and PIT with the H_3_R protein predicted their possible binding patterns and gave a presentation that **2h**, **4a**, and PIT had a similar binding model with H_3_R.

## Figures and Tables

**Table 1:** H3R antagonistic effects of targets **2a-2i**, **3a-3c**, and **4a-4b**. 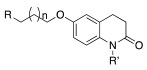

Compounds	R	*n*	R′	H_3_R Antagonistic Activity (IC_50_, μM)	ClogP ^a^
**2a**	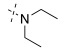	1	H	0.52	2.72
**2b**	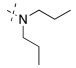	1	H	>50	3.78
**2c**	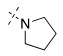	1	H	0.47	2.29
**2d**	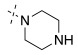	1	H	>50	1.23
**2e**	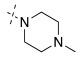	1	H	38.11	0.74
**2f**	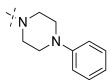	1	H	25.64	3.63
**2g**	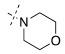	1	H	46.04	1.64
**2h**	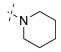	1	H	0.12	2.85
**2i**	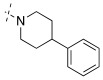	1	H	38.47	4.26
**3a**	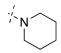	0	H	7.49	2.55
**3b**	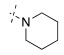	2	H	2.88	2.96
**3c**	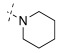	3	H	>50	3.49
**4a**	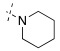	1	C_3_H_7_	0.37	3.98
**4b**	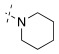	1	C_5_H_11_	1.91	5.03
**PIT**	-	-	-	0.69	4.82

**^a^** ClogP was calculated by ChemDraw 16.0 (CambridgeSoft, Cambridge, MA, USA).

**Table 2:** The neurotoxicity of compounds **2h**, **4a**, and **4b** (i.p.) in the rotarod test in mice.

Compounds	Neurotoxicity		
	10 mg/kg	30 mg/kg	100 mg/kg
**2h**	0/3	0/3	1/3
**4a**	0/3	0/3	0/3
**4b**	0/3	0/3	0/3
**PIT**	0/3	0/3	0/3

## Data Availability

Not applicable.

## Sample Availability

Samples of the compounds are not available from the authors.
